# Enhanced HER-2 prediction in breast cancer through synergistic integration of deep learning, ultrasound radiomics, and clinical data

**DOI:** 10.1038/s41598-025-12825-7

**Published:** 2025-07-24

**Authors:** Meijuan Hu, Lianying Zhang, Xiao Wang, Xuehua Xiao

**Affiliations:** Department of Ultrasound, Affiliated Hospital, Jiujiang Medical College, Jiujiang, 332000 Jiangxi China

**Keywords:** Breast cancer, HER-2, Ultrasound radiomics, Machine learning, Deep learning, Cancer, Computational biology and bioinformatics, Medical research

## Abstract

This study integrates ultrasound Radiomics with clinical data to enhance the diagnostic accuracy of HER-2 expression status in breast cancer, aiming to provide more reliable treatment strategies for this aggressive disease. We included ultrasound images and clinicopathologic data from 210 female breast cancer patients, employing a Generative Adversarial Network (GAN) to enhance image clarity and segment the region of interest (ROI) for Radiomics feature extraction. Features were optimized through Z-score normalization and various statistical methods. We constructed and compared multiple machine learning models, including Linear Regression, Random Forest, and XGBoost, with deep learning models such as CNNs (ResNet101, VGG19) and Transformer technology. The Grad-CAM technique was used to visualize the decision-making process of the deep learning models. The Deep Learning Radiomics (DLR) model integrated Radiomics features with deep learning features, and a combined model further integrated clinical features to predict HER-2 status. The LightGBM and ResNet101 models showed high performance, but the combined model achieved the highest AUC values in both training and testing, demonstrating the effectiveness of integrating diverse data sources. The study successfully demonstrates that the fusion of deep learning with Radiomics analysis significantly improves the prediction accuracy of HER-2 status, offering a new strategy for personalized breast cancer treatment and prognostic assessments.

**Supplementary Information:**

The online version contains supplementary material available at 10.1038/s41598-025-12825-7.

## Introduction

Breast cancer has become the most common type of cancer among women globally, with more than 2.3 million new cases in 2020, accounting for 11.7% of all new cancer cases, and about 685,000 deaths in 2020, accounting for 6.9% of all cancer deaths. Breast cancer has also become the leading cause of cancer deaths in 110 countries^1^. Molecular subtypes of breast cancer, such as HER-2 (Human Epidermal Growth Factor Receptor 2)-positive breast cancer, have biological behaviors and prognostic characteristics that are significantly different from those of other subtypes and, therefore, require more refined treatment and management. Approximately 20% of breast cancer cases are HER-2 positive^2,3^ and have shorter disease-free survival and relatively poor prognosis due to high proliferation rate and risk of lymph node metastasis^4^. For patients with HER-2-positive breast cancer, anti-HER-2 targeted therapy has become part of the standard treatment regimen, significantly improving the survival and quality of life of such patients. Studies have shown that the 5-, 10- and 15-year survival rates for HER-2-positive metastatic breast cancer patients treated with trastuzumab are 96%, 86%, and 60%, respectively^5^. Accurate determination of HER-2 status is essential for developing effective treatment strategies^6^.

With the continuous advancement of medical imaging technology, ultrasound imaging has become an essential tool in the diagnosis and evaluation of breast cancer. In particular, ultrasound Radiomics can reveal tumors’ biological characteristics and behavioral patterns more deeply by extracting and analyzing image features^7^. These features are not only related to visual information such as tumor morphology and texture but also reflect the microstructure and functional status of the tumor, enabling researchers to more accurately predict the degree of malignancy, molecular subtype, and response to treatment. In particular, ultrasound Radiomics shows excellent potential in predicting HER-2 expression status, which is expected to provide clinicians with more precise and personalized treatment recommendations^8,9^.

In summary, this study aims to predict the HER-2 expression status of breast cancer patients by combining machine learning and deep learning models using ultrasound Radiomics data to provide a more reliable basis for developing treatment strategies and prognostic assessment. By deeply exploring the value of ultrasound Radiomics in predicting HER-2 status, we expect to contribute new ideas and methods for precision medicine in breast cancer.

## Materials and methods

### Patient information

210 female breast cancer patients admitted to our hospital from June 2017 to October 2023 were included in this study. Inclusion criteria: (1) diagnosis of invasive ductal carcinoma confirmed by surgical resection; (2) presence of a solitary lumpy breast tumor; (3) verification of HER-2 status by biopsy/excision specimen; (4) ultrasonic examination within two weeks prior to surgery. Exclusion criteria: (1) inadequate ultrasound images or incomplete lesion capture; (2) history of radiotherapy, chemotherapy, or neoadjuvant therapy before surgery; (3) lack of comprehensive clinical information. After screening, the enrolled breast cancer patients were divided into train and test groups in a ratio of 7:3 to ensure the credibility of the prediction model.

The primary clinical and histopathological data are age, HER-2, ER, PR, Ki-67, CEA, CA125, CA199, CA153, lymphatic metastasis, calcification, menopause, family history, and ultrasound images. HER-2 expression was quantified using ASCO guidelines. Quantification of HER-2 expression included both immunohistochemistry (IHC) and in situ hybridization (FISH). IHC 0 and 1+: HER-2-, IHC 3+: HER-2+, and IHC 2+: further FISH was required to determine the amplification status of the HER-2 gene^10^. The study was conducted following the ethical guidelines of the Declaration of Helsinki^11^ and was approved by the Institutional Review Board of the Affiliated Hospital of Jiujiang Medical College (No. jjumer-a-2017-0203). Due to the retrospective nature of this study and the fact that the study did not carry out any intervention on patients, the informed consent requirement was waived by the Institutional Review Board of the Affiliated Hospital of Jiujiang Medical College, and all patient data were carefully anonymized.

### Data preprocessing

A bilateral breast ultrasonic examination was performed using PHILIPS EPIQ6, PHILIPS EPIQ5, GE Voluson E8, and Vivid E8 ultrasound systems. A linear transducer with a probe frequency range of 3–11 and 7–14 MHz was used according to the standard scanning protocol. Both longitudinal and transverse slices were acquired and saved in digital medical imaging and communication (DICOM) format for subsequent evaluation.

The clarity of ultrasound images was enhanced using a super-resolution reconstruction technique facilitated by a generative adversarial network (GAN)^12^. Employing this method, we achieve a fourfold increase in the spatial resolution of ultrasound images, significantly enhancing the accuracy of breast cancer HER-2 status predictions based on these images (Figure S1). The GAN-processed images improved Rad model AUC by 6.2% (training) and 4.1% (test). No hallucinated features were observed.

### Radiomics (Rad) procedure

#### ROI segmentation

The Region of Interest (ROI) was delineated using ITK-SNAP^13^ by two radiologists independently who were not involved in the initial ultrasound examinations (Intra-observer Correlation Coefficient (ICC) = 0.89). Two radiologists were blinded to the patients’ HER-2 status to minimize observer bias. In cases of disagreement, cases with ICC < 0.75 underwent third expert adjudication (12% of the total).

#### Feature extraction

1,561-dimensional handcrafted radiomic features were categorized into three primary groups: (1) Geometry, (2) Intensity, and (3) Texture. The gray-level co-occurrence matrix (GLCM), gray-level run length matrix (GLRLM), gray-level size zone matrix (GLSZM), and neighborhood gray-tone difference matrix (NGTDM) techniques were utilized for texture feature extraction^14^. Following the Imaging Biomarker Standardization Initiative (IBSI) guidelines, each subregion feature was extracted with the pyradiomics tool (version 3.0.1).

#### Feature selection

Feature selection was performed using a multistep approach, including Z-value normalization, p-value screening based on t-test (retaining features with *p* < 0.05), Pearson’s correlation coefficient (*r* > 0.8) to remove highly correlated features, and greedy recursive deletion strategy to reduce redundancy. Finally, the feature set was further optimized by LASSO regression, and the optimal regularization parameter λ was determined by 10-fold cross-validation. All feature selection steps were restricted to the training cohort to prevent data leakage.

#### Radiomics (Rad) model

After LASSO-based feature refinement, risk evaluation was conducted using machine learning models: linear models (LR) and tree-based models (RandomForest, ExtraTrees, XGBoost, LightGBM)^15^. For the tuning of model hyperparameters, 5-fold cross-validation on the training set was applied, and the Gridsearch algorithm was utilized to optimize the hyperparameter. The model parameters that demonstrated the best median performance were selected for the final model training.

### Deep learning (DL) procedure

#### Data preparation

The slice that presented the largest Region of Interest (ROI) for each patient was selected as the representative image. The smallest bounding rectangle encompassing the ROI was retained To reduce complexity and minimize background noise in the algorithmic analysis. This rectangle was expanded by an additional 10 pixels. Our approach standardized the intensity distribution across RGB channels through Z-score normalization of the images. These normalized images were then utilized as inputs for the model. During the training phase, real-time data augmentation strategies were implemented, including random cropping, horizontal flipping, and vertical flipping. For test images, processing was restricted to normalization only.

#### Deep learning (DL) model

Transformer technology^16^ and traditional CNN-based models (CrossFormer, ResNet50, ResNet101, VGG19, and DenseNet121) were evaluated^17^. The output probabilities generated by these models were defined as the Deep Learning (DL) Signature. The Gradient-weighted Class Activation Mapping (Grad-CAM) technique was utilized for visualization^18^. Grad-CAM highlights the activations in the final convolutional layer that are relevant to HER-2 status prediction. This visualization aids in identifying the image regions that significantly impact the model’s decision-making process, thereby offering insights into the model’s interpretability.

### Deep learning radiomics (DLR) model

The CNN that exhibited the best performance was utilized to construct the Deep Learning Radiomics (DLR) model. Features were extracted from its penultimate layer, resulting in a feature dimensionality of 2048. To mitigate the risk of overfitting associated with high dimensionality, Principal Component Analysis (PCA) was applied, compressing these features to 64 dimensions. PCA retained 95% variance (64 components), balancing dimensionality and information loss. A pre-fusion algorithm combined these 64-dimensional deep learning features with 1,561-dimensional Radiomic (Rad) features to construct the DLR model, culminating in a comprehensive 1,625-dimensional feature set. Standardized features ensured balanced contributions (Rad:52%, DL:48%). Subsequently, a methodology akin to the Radiomics (Rad) Procedure was followed for feature selection and model construction.

### Clinical use

To enhance its clinical relevance, univariable and stepwise multivariable analyses were performed on all clinical features to identify those of significance. These selected clinical features were then integrated with the deep learning (DL) model predictions, leading to the development of a Logistic Regression (LR) linear model, culminating in the creation of the Combined Model. Receiver Operating Characteristic (ROC) curves were generated to evaluate the diagnostic accuracy of models. DeLong’s test was performed to compare the difference between ROC curves. Decision Curve Analysis (DCA) was conducted to ascertain the clinical utility of our predictive models. Calibration curves and Hosmer-Lemeshow (HL) analysis were employed to evaluate the model’s calibration ability. The HL analysis involves comparing the predicted probabilities with the actual outcomes, and a lower HL statistic indicates better calibration, reflecting a closer alignment between the model predictions and observed outcomes. A nomogram was developed as the graphical representation of our final model.

### Statistical analysis

Python 3.7.12 was utilized to carry out these analyses on the OnekeyAI platform, version 3.1.8, using statsmodels version 0.13.2. The scikit-learn version 1.0.2 interface was utilized to develop machine learning models. An NVIDIA 4090 GPU, along with the MONAI 0.8.1 and PyTorch 1.8.1 frameworks, was employed for deep learning training.

## Results

### Baseline characteristics of patients

**Fig. 1:**
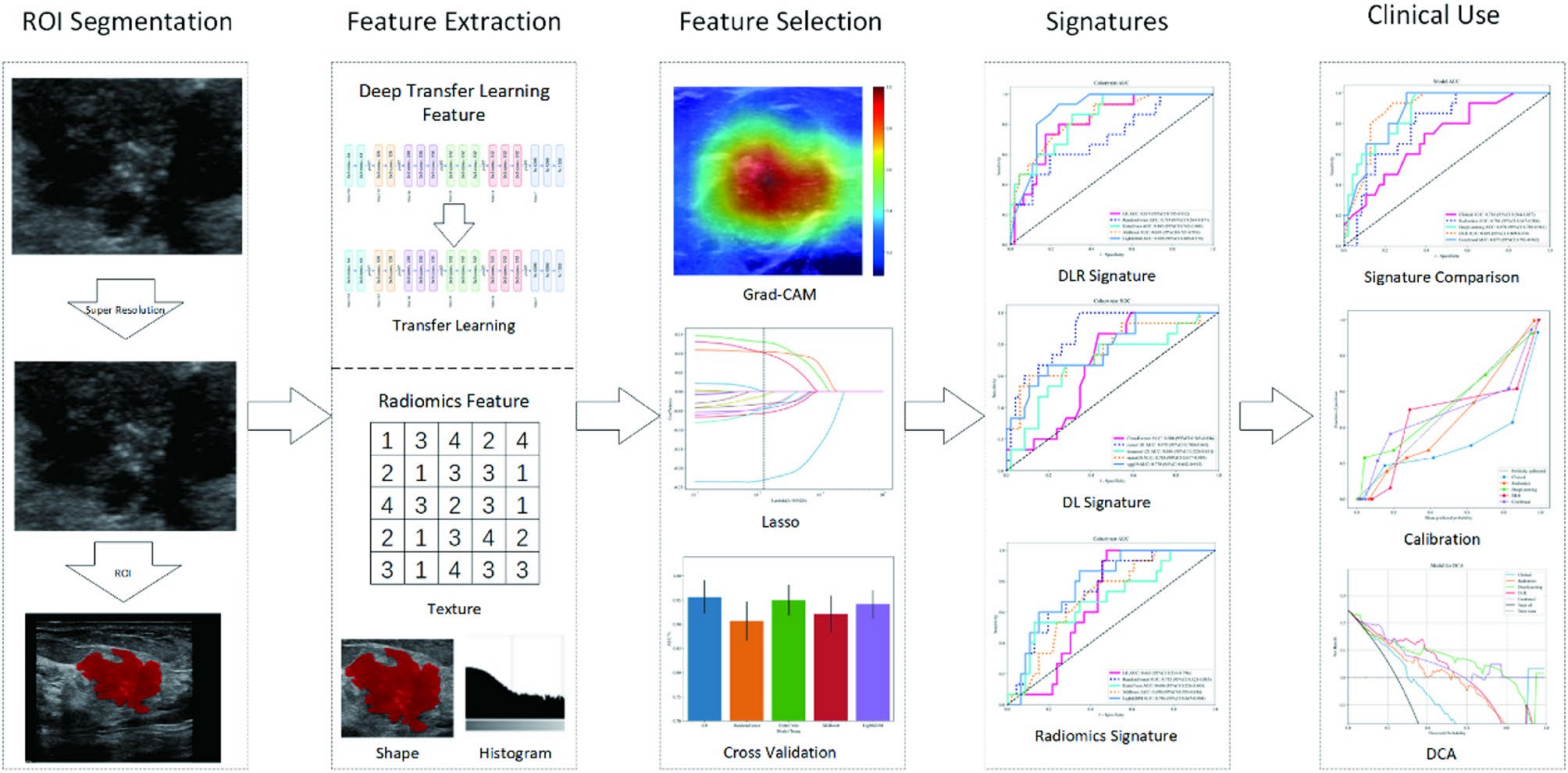
Workflow of this study.

We randomly divided the dataset, allocating 70% to the training group and 30% to the test group. Figure 1 illustrates the selection process, model establishment, and model evaluation. As depicted in Table 1, the results revealed no significant statistical differences between the training and test cohorts (p-value > 0.05), ensuring an unbiased data division.

**Table 1:** Baseline characteristic of two cohorts.

Feature name	All	Training	Test	*p*-value
Age	54.00 ± 11.04	53.36 ± 11.26	55.44 ± 10.45	0.221
Ki 67	38.78 ± 23.31	38.83 ± 22.80	38.67 ± 24.62	0.795
CEA	4.80 ± 37.12	1.98 ± 2.09	11.29 ± 67.24	0.174
CA125	65.24 ± 38.06	65.94 ± 36.87	63.63 ± 40.95	0.729
CA199	13.08 ± 34.56	12.30 ± 37.93	14.87 ± 25.38	0.42
CA153	9.19 ± 7.71	9.00 ± 4.93	9.65 ± 11.90	0.738
ER				0.418
Negative	59(29.35)	44(31.43)	15(24.59)	
Positive	142(70.65)	96(68.57)	46(75.41)	
PR				0.577
Negative	83(41.30)	56(40.0)	27(44.26)	
Positive	118(58.70)	84(60.00)	34(55.74)	
Lymphatic metastasis				0.957
No	134(66.67)	94(67.14)	40(65.57)	
Yes	67(33.33)	46(32.86)	21(34.43)	
Calcification				0.384
No	121(60.20)	81(57.86)	40(65.57)	
Yes	80(39.80)	59(42.14)	21(34.43)	
Menopausal				0.115
Pre-menopausal	91(45.27)	69(49.29)	22(36.07)	
Post-menopausal	110(54.73)	71(50.71)	39(63.93)	
Family history				0.955
No	193(96.02)	135(96.43)	58(95.08)	
Yes	8(3.98)	5(3.57)	3(4.92)	

*Ki-67* Ki-67 protein, *CEA* Carcinoembryonic Antigen, *CA125* Cancer Antigen 125, *CA199* Cancer Antigen 199, *CA153* Cancer Antigen 153, *ER* Estrogen Receptor, *PR* Progesterone Receptor.

### Clinical features analysis

Univariable and multivariate analysis of all clinical features described the association between clinical features and HER-2 expression status. PR was chosen for inclusion in the nomogram construction (Table 2).

**Table 2:** Univariable and multivariable analysis of clinical features.

	Univariable analysis				Multivariable analysis			
Feature name	OR	lower 95%CI	upper 95%CI	p-value	OR	lower 95%CI	upper 95%CI	p-value
ER	0.263	0.174	0.398	0	0.601	0.224	1.614	0.397
Menopausal	0.479	0.316	0.727	0.004	1.277	0.548	2.977	0.634
Lymphatic metastasis	0.533	0.32	0.888	0.042	1.062	0.516	2.184	0.891
PR	0.538	0.443	0.654	0	0.343	0.151	0.778	0.032
Family history	0.667	0.149	2.992	0.657				
Calcaification	0.686	0.444	1.061	0.155				
CEA	0.775	0.674	0.891	0.003	1.066	0.91	1.247	0.506
CA153	0.935	0.908	0.965	0	1.04	0.969	1.116	0.356
CA199	0.95	0.923	0.978	0.003	0.996	0.977	1.015	0.716
Age	0.985	0.98	0.991	0	0.995	0.969	1.022	0.77
Ki 67	0.99	0.983	0.996	0.011	1.009	0.994	1.025	0.33
CA125	0.991	0.987	0.995	0	1.006	0.997	1.015	0.265

*Ki-67* Ki-67 protein, *CEA* Carcinoembryonic Antigen, *CA125* Cancer Antigen 125, *CA199* Cancer Antigen 199, *CA153* Cancer Antigen 153, *ER* Estrogen Receptor, *PR* Progesterone Receptor, *OR* Odds Ratio, *CI* Confidence Interval.

**Fig. 2:**
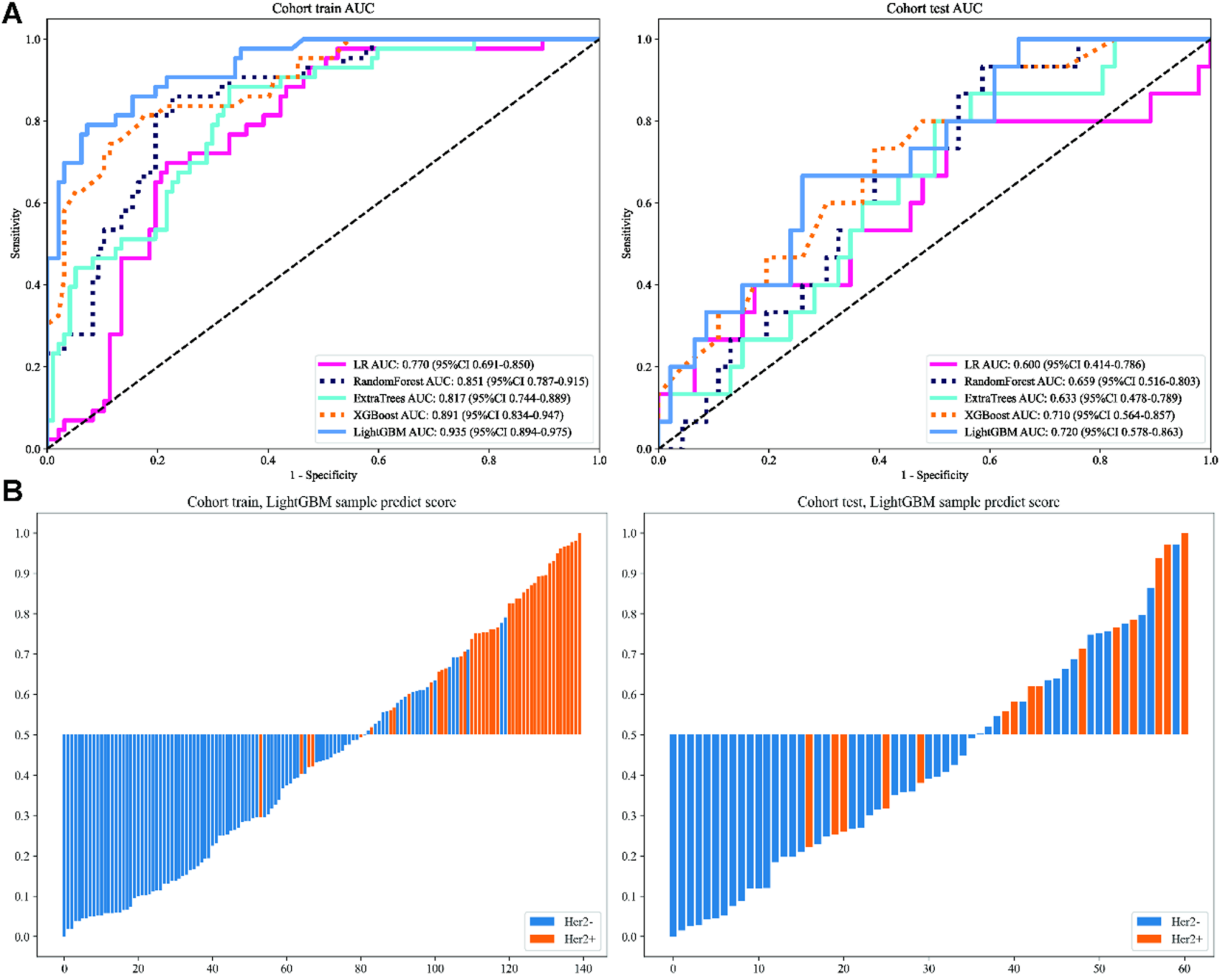
ROC curves (**A**) and prediction histogram (**B**) for the clinical model. Left panel: train cohort; right panel: test cohort.

### Clinical model

In order to predict the expression status of HER-2, We numerically mapped all clinical features and then modeled these features using machine learning algorithms (linear models (LR) and tree-based models (random-forest, ExtraTrees, XGBoost, LightGBM)). In evaluating the performance of various predictive models for our study, the LightGBM algorithm demonstrated superior efficacy according to the Area Under the Curve (AUC). LightGBM achieved an AUC of 0.935 (95% CI: 0.894–0.975) on the training cohort and 0.720 (95% CI: 0.578–0.863) on the test cohort, indicating a high level of diagnostic accuracy. This model (Table S1, Fig. 2A) not only outperformed Logistic Regression (LR), Random Forest, ExtraTrees, and XGBoost but also maintained robustness in sensitivity (76.7% in the training cohort, 60% in the test cohort) and specificity (92.8% on the training cohort, 73.9% on the test cohort). The sample prediction histogram also intuitively shows the prediction results of each sample and the corresponding actual results (Fig. 2B). Given these results, LightGBM was selected for downstream signature comparison studies, a decision that was based on its demonstrated predictive strength and reliability across diverse patient samples.

### Radiomics (Rad) model

**Fig. 3:**
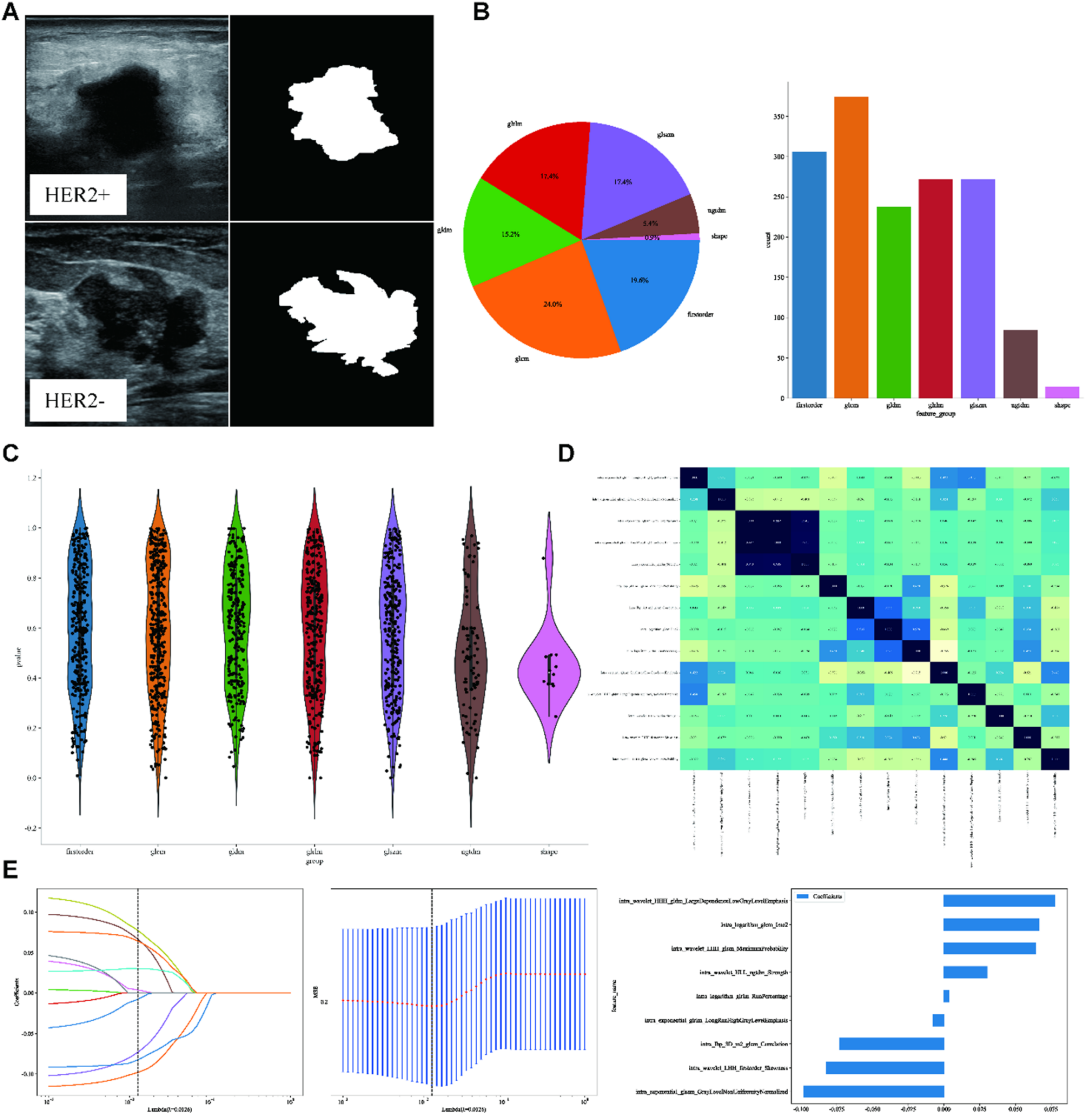
ROI Segmentation (**A**), Feature Extraction (**B**), Feature Selection (CDE) in Radiomics (Rad) Procedure. (**A**) Intratumoral ROIs in ultrasound imaging for two typical samples, identified as “HER2+” and “HER2-“: grayscale US image (left) and corresponding mask (right). (**B**) Pie charts (left) and histograms (right) show numbers and ratios of handcrafted features. (**C**) The violin chart displays the p-value screening using a t-test (retaining features with *p* < 0.05) for different features. (**D**) Pearson’s Correlation matrix shows the correlation coefficient of handcrafted features. (**E**) LASSO regression. The sub-figures correspond to the Lasso path, Mean Squared Error (MSE), and weights of the selected radiomic features.

The Region of Interest (ROI) delineation was carried out independently using ITK-SNAP by two experienced radiologists. Figure 3A illustrates intratumoral ROIs in BC ultrasound imaging. In order to avoid observer bias, the radiologists responsible for ROI segmentation were independent of the ultrasound operators and blinded to HER-2 status. This design ensures that segmentation decisions are based solely on imaging features, reducing confounding from clinical knowledge. A comprehensive 1561 handcrafted radiomic features were extracted and classified into three primary categories: shape, first-order, and texture, including 360 first-order features, 14 shape features, and diverse texture features. The distribution of these handcrafted features across the various categories is visually depicted in Fig. 3B. The violin chart displays the p-value screening based on a t-test (retaining features with *p* < 0.05) for different features (Fig. 3C). Pearson’s correlation coefficient removes highly correlated features, and a greedy recursive deletion strategy is used to reduce redundancy (Fig. 3D). The radiomics features were selected using a Lasso-based method with 10-fold cross-validation, as depicted in Fig. 3E.

**Fig. 4:**
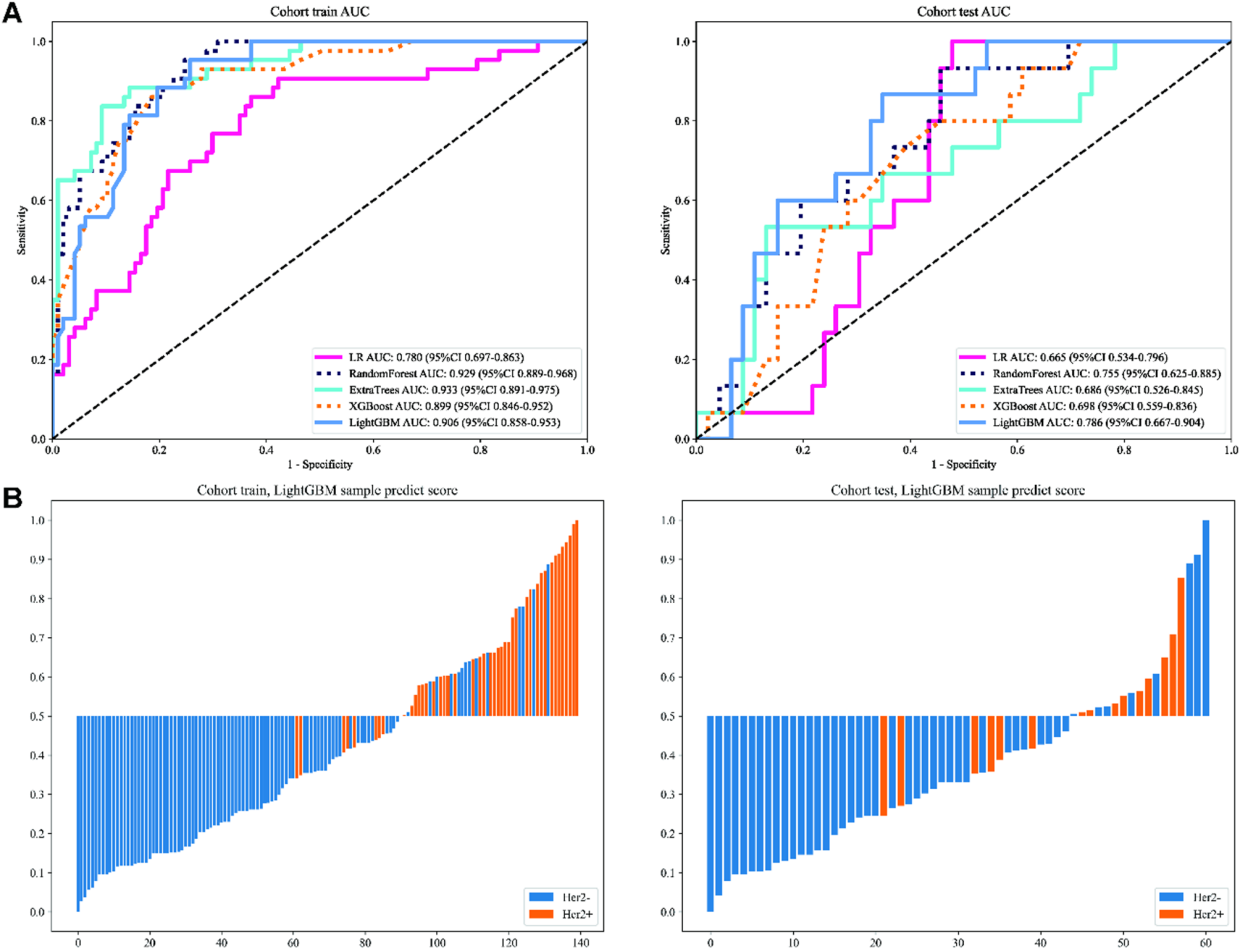
ROC curves (**A**) and prediction histogram (**B**) for the Radiomics (Rad) model. Left panel: train cohort; right panel: test cohort.

We numerically mapped all selected Rad features and then modeled these features using machine learning algorithms similar to those employed in the clinical Signature. LightGBM achieved an AUC of 0.906 (95% CI: 0.858–0.953) on the training cohort and 0.786 (95% CI: 0.667–0.904) on the test cohort, indicating a high level of diagnostic accuracy. This model maintained robustness in sensitivity (93.0% in the training cohort, 80% in the test cohort) and specificity (74.2% in the training cohort, 65.2% in the test cohort). Figure 4A presents figures corresponding to the models’ performance on Rad Signature training and test datasets (Table S2). The sample prediction histogram shows the prediction results of each sample and the corresponding actual results (Fig. 4B).

### Deep learning radiomics (DLR) model

#### Deep learning (DL) model

**Fig. 5:**
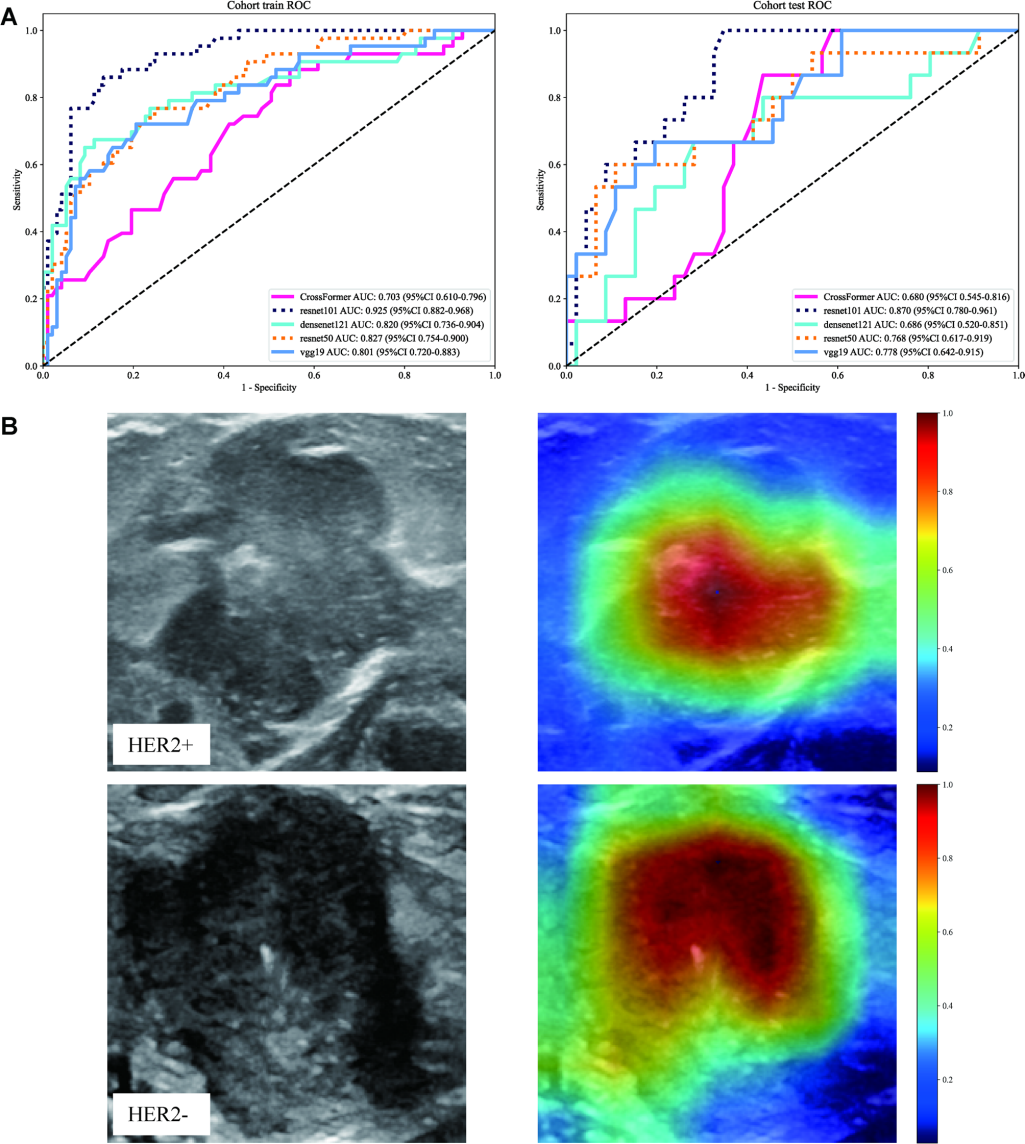
ROC curves (**A**) and Grad-CAM visualizations (**B**) for the Deep Learning (DL) model. (**A**) ROC curves of Deep Learning model. Left panel: train cohort; right panel: test cohort. (**B**) Grad-CAM visualizations for two typical samples, identified as"HER2+” and"HER2-“.

The output probabilities generated by Transformer technology and traditional CNN-based models (CrossFormer, ResNet50, ResNet101, VGG19, and DenseNet121) were defined as the Deep Learning (DL) Signature. In our analysis of various deep learning models, the ResNet101 model emerged as the most effective (Table S3, Fig. 5A), demonstrating the highest AUC of 0.925 (95% CI: 0.8820–0.9679) in the training cohort and 0.870 (95% CI: 0.7801–0.9605) in the test cohort. These results, coupled with its robust sensitivity and specificity, substantiate our choice of using the ResNet101 model for feature extraction and Grad-CAM visualizations (Fig. 5B). In the test cohort, True Positives (TP) were 19 cases (HER-2 + correctly classified), True Negatives (TN) were 24 cases (HER-2- correctly classified), False Positives (FP) were 9 cases (HER-2- incorrectly classified as HER-2+), False Negatives (FN) were 11 cases (HER-2 + incorrectly classified as HER-2-). Grad-CAM visualizations revealed that in some FP cases, the model highlighted areas with high intensity due to calcification or lymph node metastasis, which are not directly associated with HER-2 status. The model may be influenced by non-specific features that resemble tumor characteristics in specific contexts. Some FN cases showed that the model’s attention was dispersed across the image, missing the subtle texture patterns within the tumor that are indicative of HER-2 positivity. The complexity and variability of tumor textures in ultrasound images may sometimes be overlooked. Although there are a few FP and FN cases, Grad-CAM still highlights the activations in the final convolutional layer relevant to HER-2 status prediction, helps identify image regions that significantly impact the model’s decision-making, and offers insights into its interpretability.

#### Deep learning radiomics (DLR) model

**Fig. 6:**
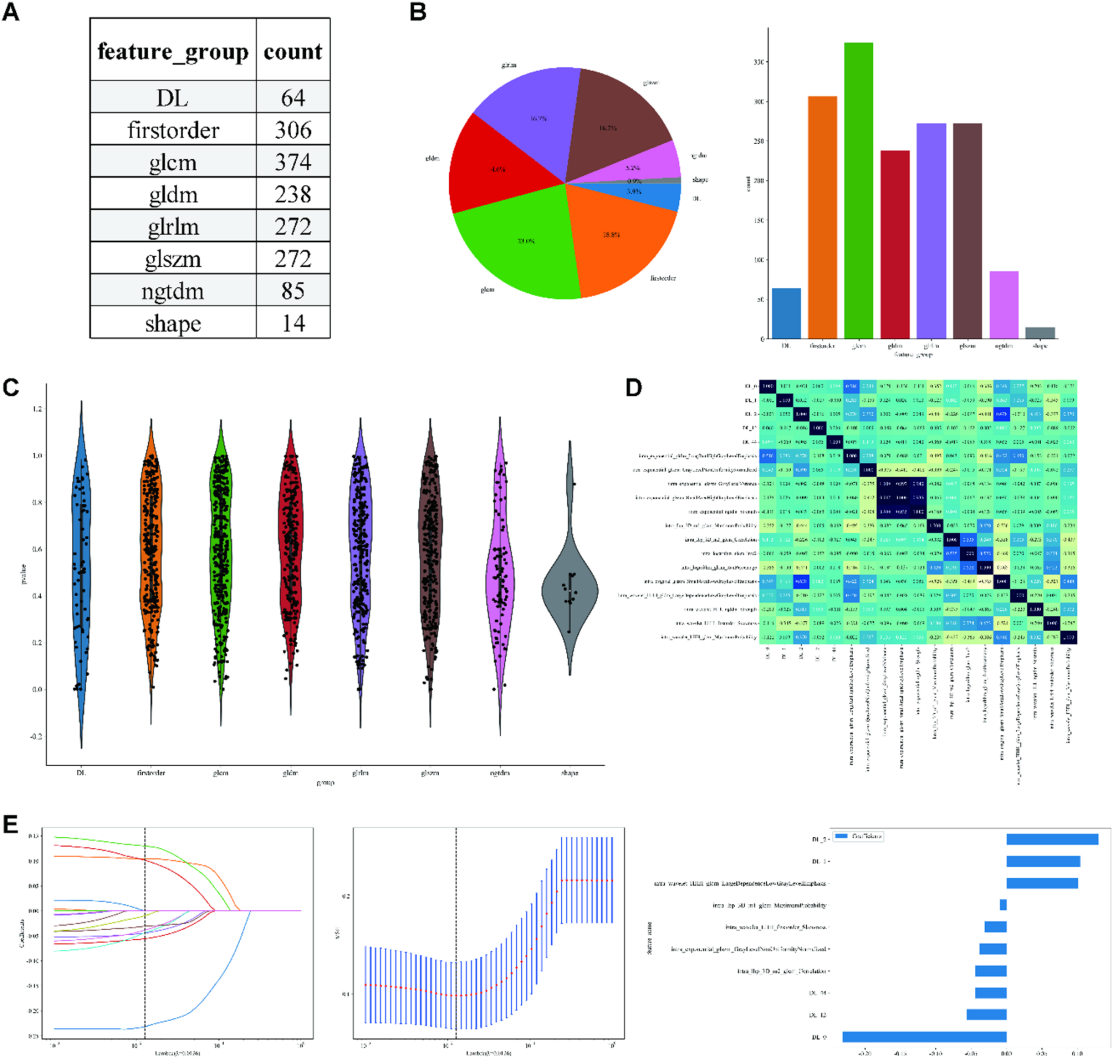
Deep Learning Radiomics (DLR) Procedure. (**A**) 64 DL features combined with 1,561 Rad features to construct the DLR model. (**B**) Pie charts (left) and histograms (right) show numbers and ratios of DLR features. (**C**) The violin chart displays the p-value screening using a t-test (retaining features with *p* < 0.05). (**D**) Pearson’s Correlation matrix shows the correlation coefficient of DLR features. (**E**) LASSO regression. The sub-figures correspond to the Lasso path, Mean Squared Error (MSE), and weights of the selected radiomic features.

**Fig. 7:**
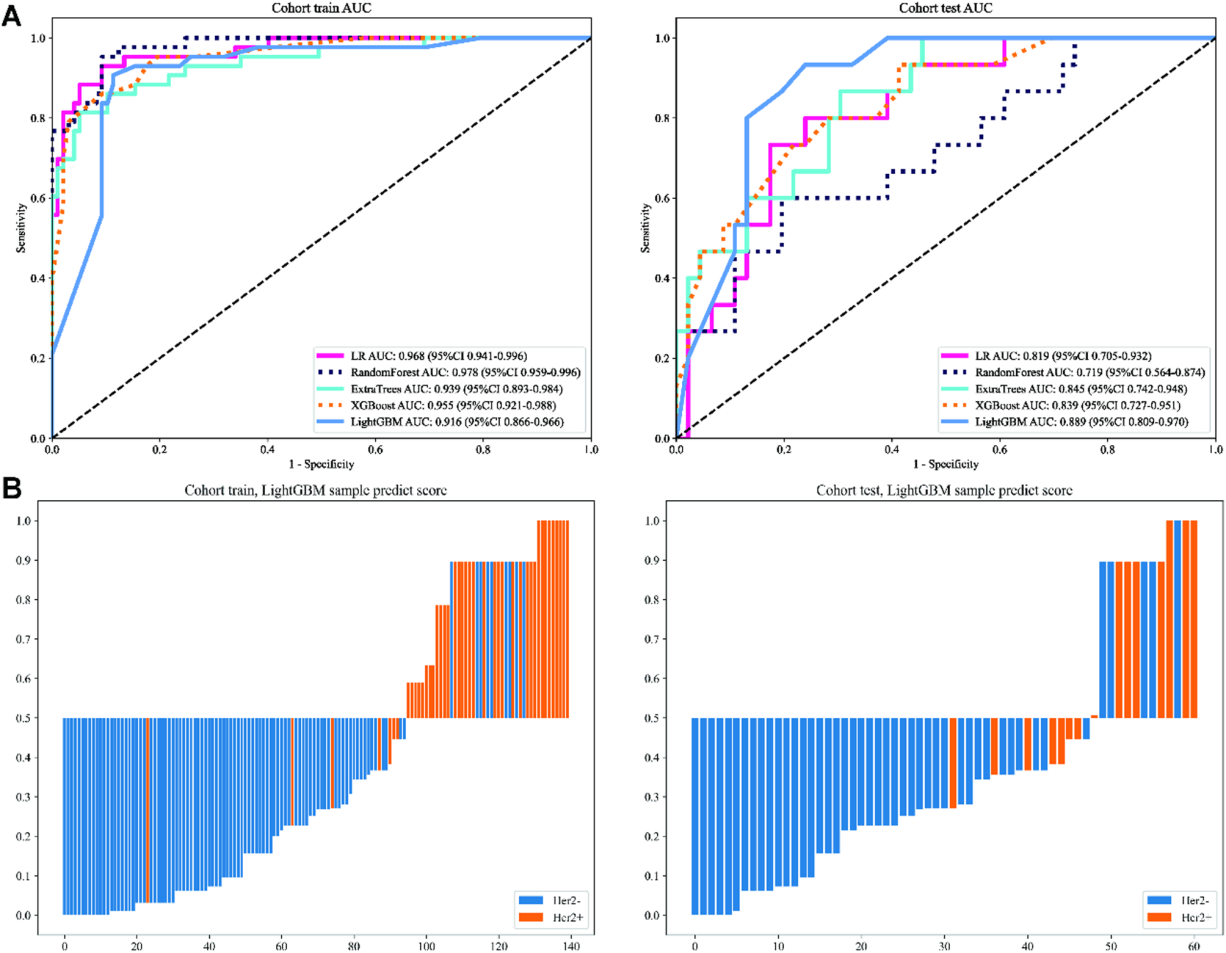
ROC curves (**A**) and prediction histogram (**B**) for Deep Learning Radiomics (DLR) model. Left panel: train cohort; right panel: test cohort.

The DLR model was constructed using the top-performing CNN’s penultimate layer, initially providing a 2048-dimensional feature set. PCA was applied to compress this set to 64 dimensions, mitigating the risk of overfitting. Subsequently, a pre-fusion algorithm combined 64 DL features with 1,561-dimensional Rad features, creating a 1,625-dimensional feature set (Fig. 6AB). The feature selection and model construction process is akin to that of Rad Procedure. The violin chart displays the p-value screening based on t-test (retaining features with *p* < 0.05) for different features (Fig. 6C). Pearson’s correlation coefficient removes highly correlated features, and a greedy recursive deletion strategy is used to reduce redundancy (Fig. 6D). The radiomics features were selected using a Lasso-based method with 10-fold cross-validation, as depicted in Fig. 6E. We numerically mapped all selected DLR features and then modeled these features using machine-learning algorithms similar to those employed in the clinical signature. DLR model (LightGBM) achieved an AUC of 0.916 (95% CI: 0.866–0.966) in the training cohort and 0.889 (95% CI: 0.809–0.970) in the test cohort, indicating a high level of diagnostic accuracy. DLR model robustness in sensitivity (88.4% in the training cohort, 86.7% in the test cohort) and specificity (88.7% in the training cohort, 80.4% in the test cohort), which underscores the synergistic effect of integrating deep learning and radiomic features, which harnesses the strengths of both modalities to capture the complexities of the data better. Figure 7A presents figures corresponding to the performance of the models on DLR signature training and test datasets (Table S4). The sample prediction histogram shows the prediction results of each sample and the corresponding actual results (Fig. 7B).

### Clinical use

**Fig. 8:**
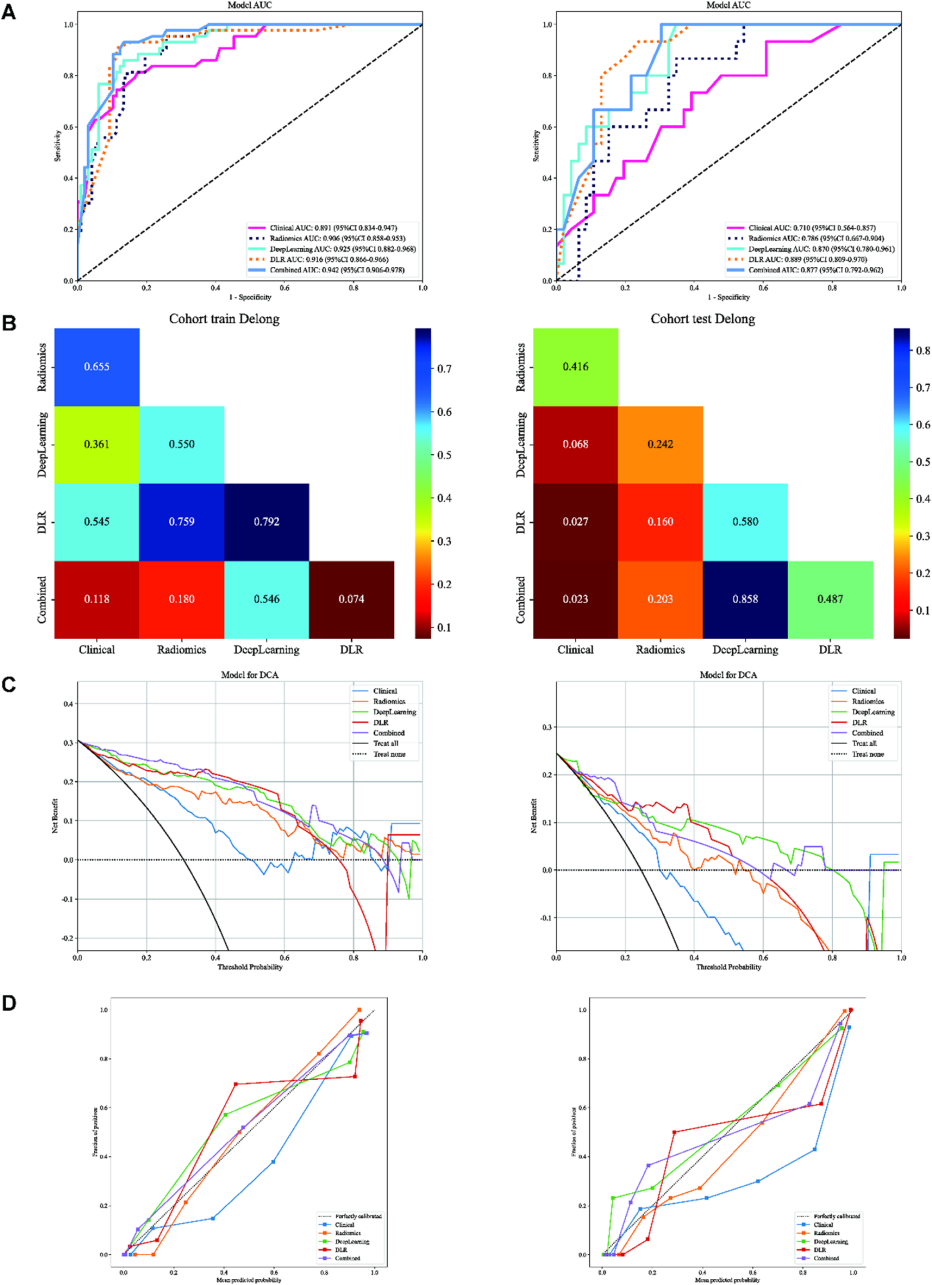
Clinical Use of Combined Model. (**A**) ROC curves for different models. (**B**) DeLong’s test of AUC for different models. (**C**) DCA for different models. (**D**) Calibration curves for different models. Left panel: train cohort; right panel: test cohort.

The Combined model, which integrates the clinical model (result from 3.3) with the DLR model (result from 3.5.2), showed an improvement in the training cohort (Table S5, Fig. 8A left panel) with an AUC of 0.942 (95% CI: 0.9065–0.9782).

There is no statistically significant difference between these models, showing Clinical, Rad, DL, DLR, and combined models all have high prediction efficiency in training cohorts (Fig. 8B left panel, *p* > 0.05, DeLong’s test). However, this improvement was not replicated in the test cohort (Fig. 8A right panel), where the AUC (0.877, 95% CI: 0.7918–0.9618) was slightly lower than that of the DLR model (AUC = 0.889, 95% CI: 0.809–0.970). DeLong’s test shows no statistically significant difference between these two models (Fig. 8B right panel, p-value = 0.487, DeLong’s test), suggesting that the addition of clinical features did not contribute significant additional informational value beyond what was provided by the DLR model. Subsequently, we compared the predictive performance of the Combined model with other models using Decision Curve Analysis (DCA). These curves reveal that our fusion model provides considerable net benefit advantages regarding HER-2 predictive probabilities (Fig. 8C). Calibration curves and HL analysis show that the Combined model demonstrated excellent calibration. The HL test statistic was 0.071 for the Combined model in the test cohort (Fig. 8D right panel), indicating effective calibration, thus accurately confirming their reliability in mirroring observed data.

**Fig. 9:**
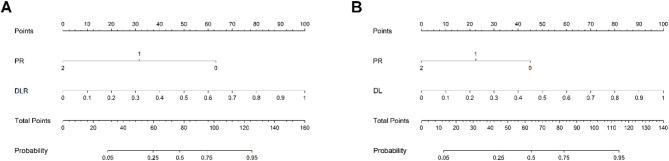
Comprehensive nomograms for predicting HER-2 status in breast cancer.

After that, three sets of factors (PR status or clinical model, Rad model, DL model, or DLR model) were included in the nomogram development to assist in predicting HER-2 status. Due to the inclusion relationship between PR status and clinical model, DL model and DLR model, only one of these two sets of factors can be selected for inclusion in the nomogram. Moreover, the Rad model was excluded because of the non-statistically significant contribution (*p* > 0.05). Figure 9 displayed the probability of correctly predicting HER-2 expression status.

## Discussion

Current innovations in precision medicine techniques guide individualized lift-step therapy for breast cancer^19,20^. A combination of local (including surgery and radiotherapy) and systemic (endocrine therapy, chemotherapy, targeted therapy, and immunotherapy) treatments gives hope for the curability of early-stage breast cancer patients^21^. However, advanced metastatic breast cancer is still considered incurable, and treatment should be individualized according to the molecular type and markers^22,23^. According to the different levels of hormone receptors and HER-2, breast cancer can be classified into different subtypes^24^. HER-2, or c-erbB2, is an epidermal growth factor receptor (EGFR) family member. HER-2 + is one of the most aggressive subtypes of breast cancer. HER-2 + breast cancers are more susceptible to metastasis (particularly metastasis to the central nervous system and viscera) and are a crucial risk factor for a diagnosis of metastatic breast cancer (stage IV), leading to poorer clinical outcomes and prognosis^25^and is, therefore, an important therapeutic target in breast cancer. The emergence of anti-HER-2 therapy has dramatically improved the prognosis of HER-2 + patients, ranging from monoclonal antibodies^26^、small molecule targeted drugs to antibody-drug conjugates^27^which have improved the pathologic complete response rate and long-term efficacy and have continuously rewritten the treatment landscape of HER-2 + advanced breast cancer treatment landscape.

This study aimed to improve the prediction accuracy of HER-2 expression status in breast cancer patients by integrating clinical features, radiomics (Rad), and deep learning (DL) techniques. In the clinical characterization, we found that although univariate analyses showed multiple factors were associated with HER-2 expression status, multivariate analyses only confirmed the statistical significance of PR status, which may imply that there is a degree of redundancy in the available set of clinical features or that the biological association of these features with HER-2 status is not as strong as expected. Various biological processes, including the tumor microenvironment, genetic heterogeneity, and individual patient differences, may influence the complex relationship between clinical features and molecular subtypes. For example, the prominence of PR status may be related to its role in the hormone receptor signaling pathway in breast cancer, which may intersect or interact with HER-2 status.

Ultrasound Radiomics technology, as an emerging non-invasive diagnostic tool, provides new perspectives and methods for diagnosing and treating breast cancer by analyzing many features in the ultrasound image^28,29^. Based on 1,561 dimensions of features extracted from ultrasound images, the Rad model constructed in this study underwent feature selection and optimization and ultimately achieved an AUC value of 0.906 in the training cohort and maintained an AUC value of 0.786 in the test cohort, showing high diagnostic accuracy. This result is consistent with previous studies and further confirms the effectiveness of Ultrasound Radiomics in breast cancer diagnosis^30–32^. Ultrasound Radiomics features may be associated with tumor aggressiveness^33^metastatic potential^34^and responsiveness to therapy^35,36^. This study used a multistep feature selection method to improve the model’s predictive accuracy and clinical utility. Although the AUC values of the test cohort were slightly lower than those of the training cohort, the model still showed good generalization capacity.

Deep learning techniques have significantly progressed in medical image analysis, especially in diagnosing^37,38^ and molecular subtype classification^39^. In this study, the deep learning models, especially ResNet101, demonstrated excellent performance, with AUC values of 0.925 and 0.870 in predicting HER-2 expression states, respectively, a result that not only confirms the potential of deep learning techniques in ultrasound image analysis but also agrees with existing studies on the high efficiency of deep learning models in medical image processing^40^. However, it is worth noting that although the Vision Transformer architecture performs well in natural image processing tasks^41^it did not achieve the expected performance on the dataset of this study, suggests that despite the advantages of the ViT architecture in processing data with long-range dependencies, traditional convolutional neural networks (CNNs) may be better suited for processing localized features and spatial hierarchies in medical image analysis. The reason may be related to the specific nature of medical image data, such as image normalization, noise levels, and technical variability in the image acquisition process^42^. In addition, the decision-making process of deep learning models is often considered a “black-box”, but the Grad-CAM technique used in this study provides a visualization tool that helps explain the model’s predictive decisions. The Grad-CAM technique enhances the model’s credibility and promotes clinicians’ acceptance of the model.

By combining DL features with Rad features, our constructed DLR model achieves AUC values of 0.916 and 0.889 in the training and test cohorts, respectively, showing good diagnostic performance and robustness. This result suggests that integrating different data models can capture the complexity of the data more effectively and thus improve the prediction accuracy. This result, together with previous studies, affirms the significant efficacy of the DLR model in several areas related to breast cancer, including, but not limited to, assessment of lymph node status^43,44^prognostic assessment^45^prediction of chemotherapeutic response^46^biomarker discovery^47^and tumor marker detection^48^. By integrating the Combined Model of clinical features and DLR models for prediction, we expected to be able to take advantage of different data sources and enhance the diagnostic capability of the model. The results showed that in the training cohort, the combined model showed better performance with an AUC of 0.942, and in the test cohort, its AUC was 0.877, slightly lower than that of the DLR model of 0.889. The clinical features and HER-2 status may be variable in different patient populations, and clinical features have not introduced new discriminative information capable of enhancing the model’s performance, thus not significantly impacting the model’s generalization capacity. The results caution against expecting consistent performance improvements across different data sets without substantial evidence of their incremental value. However, the high AUC values shown in this result and their specificity and sensitivity can also highlight the potential of the DLR and combined model in enhancing predictive accuracy.

Confusion Matrix for Combined Model (20TP, 28TN, 14FP, 1FN) demonstrates significant clinical utility in managing HER-2 status prediction in breast cancer patients. For true positives, the model enables early identification of HER-2 + patients, allowing for timely initiation of targeted therapy, which is crucial for improving prognosis and survival rates. However, the risk of false positives necessitates caution, as it may lead to overtreatment of HER-2- patients with unnecessary targeted therapy, potentially causing avoidable side effects. Clinical evaluation should complement model predictions before treatment decisions to mitigate side effects. True negatives benefit from avoiding unnecessary targeted therapy and adhering to standard treatment protocols without additional interventions. False negatives, though fewer in number, underscore the importance of integrating model predictions with clinical expertise and further diagnostic tests to ensure no patient in need of targeted therapy is overlooked. Overall, the model serves as a valuable adjunct to clinical decision-making, enhancing the precision of HER-2 status prediction while emphasizing the necessity of a balanced approach that combines algorithmic insights with professional medical judgment.

This study combined clinical information and fully applied machine learning and deep learning algorithms for HER-2 status prediction compared with previous studies. Both DLR and Combined Models showed good diagnostic performance and robustness. However, this study has some limitations. Firstly, the model’s performance on the test cohort was slightly degraded, suggesting that we need to pay attention to the ability of the dataset to generalize. Second, the Grad-CAM technique provides a certain degree of visualization and interpretation, but the “black-box” nature of deep learning models still requires further improvement in model transparency and interpretation. In addition, the single-center cohort may limit generalizability. Further clinical trials and multicenter validation are needed to assess the utility and generalizability of these models in different healthcare facilities.

## Conclusion

In this study, we successfully improved the prediction accuracy of HER-2 expression status in breast cancer patients by innovatively combining deep learning techniques and radiomics analysis. Constructed models demonstrated excellent diagnostic performance on both training and test datasets. It validates the effectiveness of multimodal data fusion in improving prediction accuracy and provides a new strategy and scientific basis for individualized breast cancer treatment. With this integrated approach, we expect to be able to provide clinicians with more accurate diagnostic tools to develop more personalized and effective treatment plans for patients.

## Supplementary Information

Below is the link to the electronic supplementary material.

Supplementary Material 1

## Data Availability

Data availability: All data generated or analysed during this study are included in this published article and its supplementary information files.
