# Supplementary material for: Enhanced HER-2 prediction in breast cancer through synergistic integration of deep learning, ultrasound radiomics, and clinical data
Source: Sci Rep. 2025 Jul 24;15:26992. doi: 10.1038/s41598-025-12825-7 (PMC12289904; doi:10.1038/s41598-025-12825-7)
Supplement: Supplementary file 1 — Supplementary Material 1 [file 41598_2025_12825_MOESM1_ESM.zip › supplementary file/revised Figure S1.pdf]

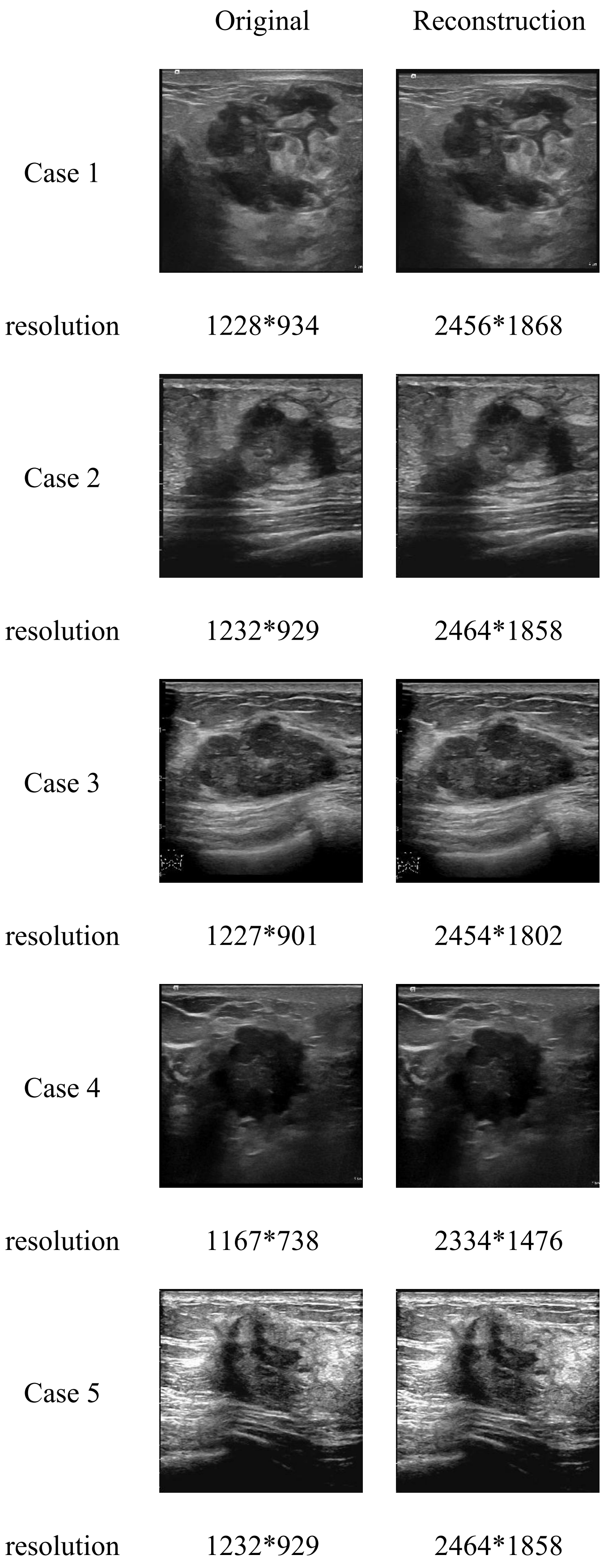

Figure S1 Super Resolution Reconstruction: 5 pairs of typical images exhibit enhanced clarity and finer details.
